# Bedside greater occipital nerve block with bupivacaine for the treatment of recalcitrant scalp pruritus

**DOI:** 10.1016/j.jdcr.2023.12.010

**Published:** 2024-01-01

**Authors:** Anusha Kambala, Alexander L. Kollhoff, Elena Wei, Kevin K. Lee, Hannah Cornman, Emily Z. Ma, Jaya Manjunath, Brenda Umenita Imo, Sriya V. Reddy, Shawn G. Kwatra

**Affiliations:** a Department of Dermatology, Johns Hopkins University School of Medicine, Baltimore, Maryland; b Department of Oncology, Johns Hopkins University School of Medicine, Baltimore, Maryland

**Keywords:** bupivacaine, chronic itch, chronic pruritus, greater occipital nerve block, local anesthetic, nerve block, neuropathic itch, neuropathic pruritus, peripheral nerve block, scalp dysesthesia, scalp pruritus

## Introduction

Scalp pruritus is commonly associated with cervical spine disease and may be accompanied by burning or stinging sensations.^1^ Scalp pruritus in these cases is considered a subtype of neuropathic pruritus (NP), a form of chronic itch caused by neuronal damage within the somatosensory system.^2^ Despite its severe impact on quality of life (QOL), NP currently has no United States Food and Drug Administration (FDA)-approved therapeutic options.^3^

Bupivacaine is a long-acting local anesthetic that is currently FDA-approved in 0.25% and 0.5% concentrations for use in peripheral nerve blocks (PNBs). PNBs are widely used in the prevention and management of various types of pain, including neuropathic pain. We thus hypothesized that PNBs may also be effective in treating NP. There have been rare reports of NP treated with CT-guided nerve block and nerve ablation, but outpatient bedside PNBs have not been reported to our knowledge.^4^^,^^5^

Here, we discuss 2 patients with recalcitrant scalp pruritus who responded well to outpatient bedside bilateral greater occipital nerve block (GONB) with 6 mL of 0.25% bupivacaine, with itch relief lasting 3 to 4 weeks.

## Case reports

### Case 1

**Fig 1:**
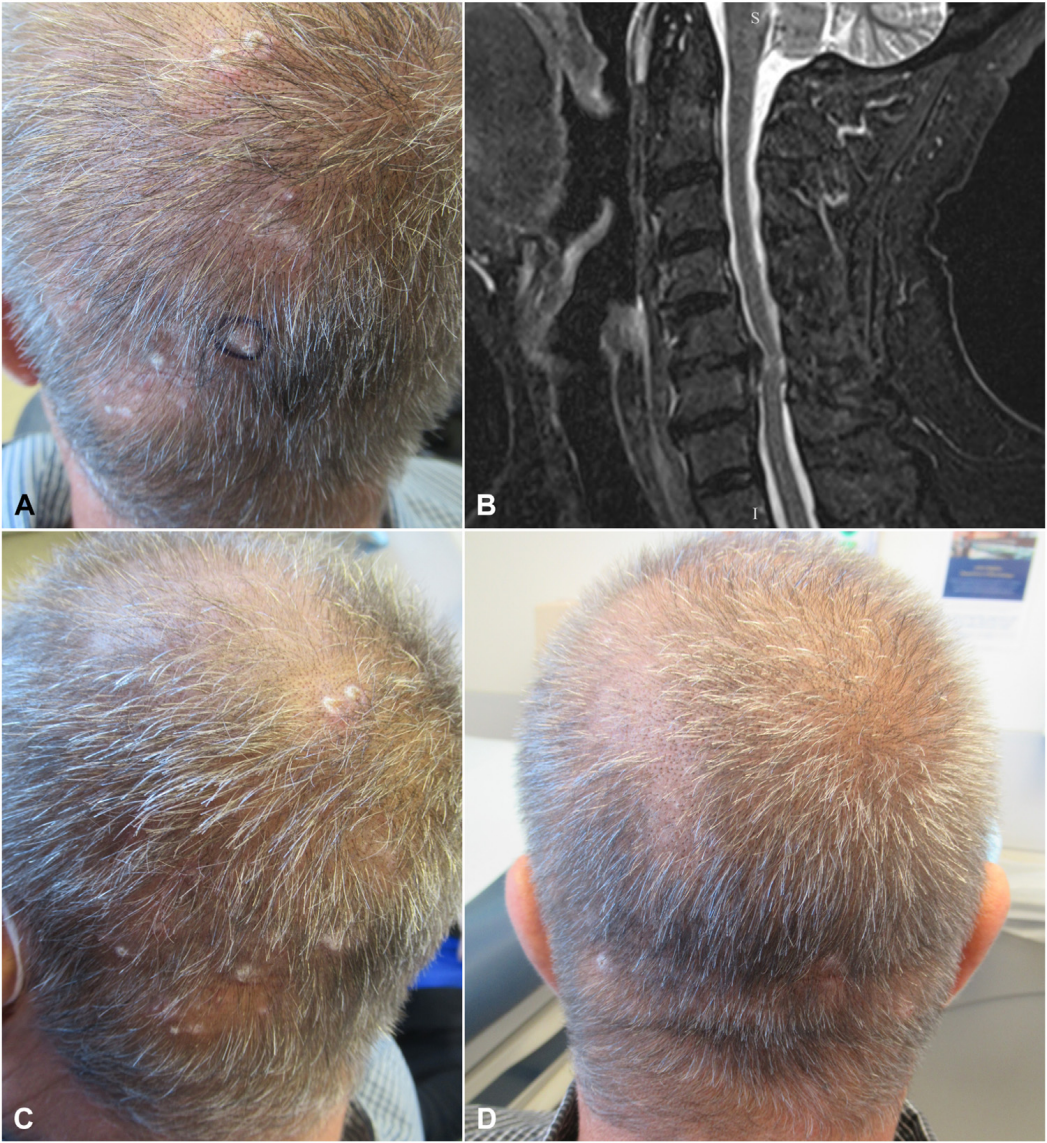
Case 1 clinical photographs and imaging. **A,** Scattered thin papules and plaques with excoriation and mild scale on the occipital scalp. **B,** MRI of the cervical spine demonstrated disc degeneration and spondylolisthesis contributing to multilevel stenosis, worst at C5-C6 where a disc herniation was associated with severe central canal stenosis and abnormal cord signal. Sagittal short tau inversion recovery (STIR) sequence is shown. **C,** Patient’s scalp prior to GONB. **D,** Patient’s scalp following the third GONB, which was performed 3 months after the first procedure. *GONB*, Greater occipital nerve block.

A 67-year-old man presented with a 3-year history of intractable occipital scalp pruritus. Itch intensity was rated as a 7/10 on the Worst Itch Numeric Rating Scale (WI-NRS), which ranges from 0 (no itch) to 10 (worst imaginable itch). Physical examination revealed scattered thin papules and plaques with excoriation and mild scale on the occipital scalp (Fig 1, *A*). Cervical magnetic resonance imaging (MRI) demonstrated disc degeneration and spondylolisthesis contributing to multilevel cervical stenosis (Fig 1, *B*). Over the next 12 months, the patient’s pruritus remained uncontrolled despite the use of numerous local and systemic agents, including intralesional steroid injections, topical corticosteroids, topical tacrolimus, topical roflumilast, naltrexone, tapinarof, excimer laser, capsaicin cream, oral methotrexate, dupilumab, gabapentin, and topical ruxolitinib.

**Fig 2:**
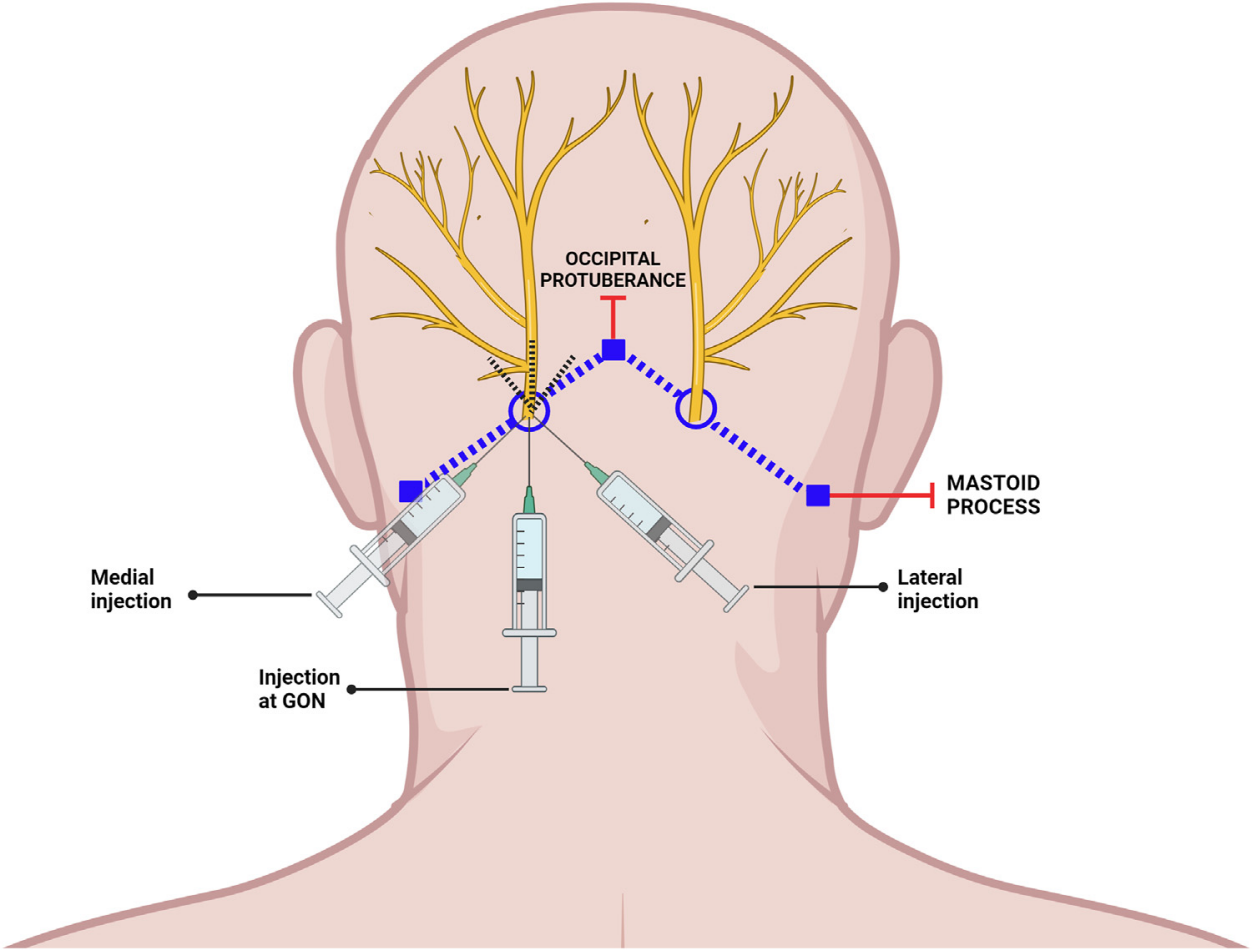
Schematic of GONB technique. The patient’s occipital protuberance and bilateral mastoid processes were identified as landmarks. The *left* injection site was located one-third of the distance from the occipital protuberance to the *left* mastoid process. Using a fanning technique, 1 mL of 0.25% bupivacaine was injected with the needle directed superiorly, 1 mL with the needle directed slightly medially, and 1 mL with the needle directed slightly laterally. The procedure was then repeated on the *right* side.

**Fig 3:**
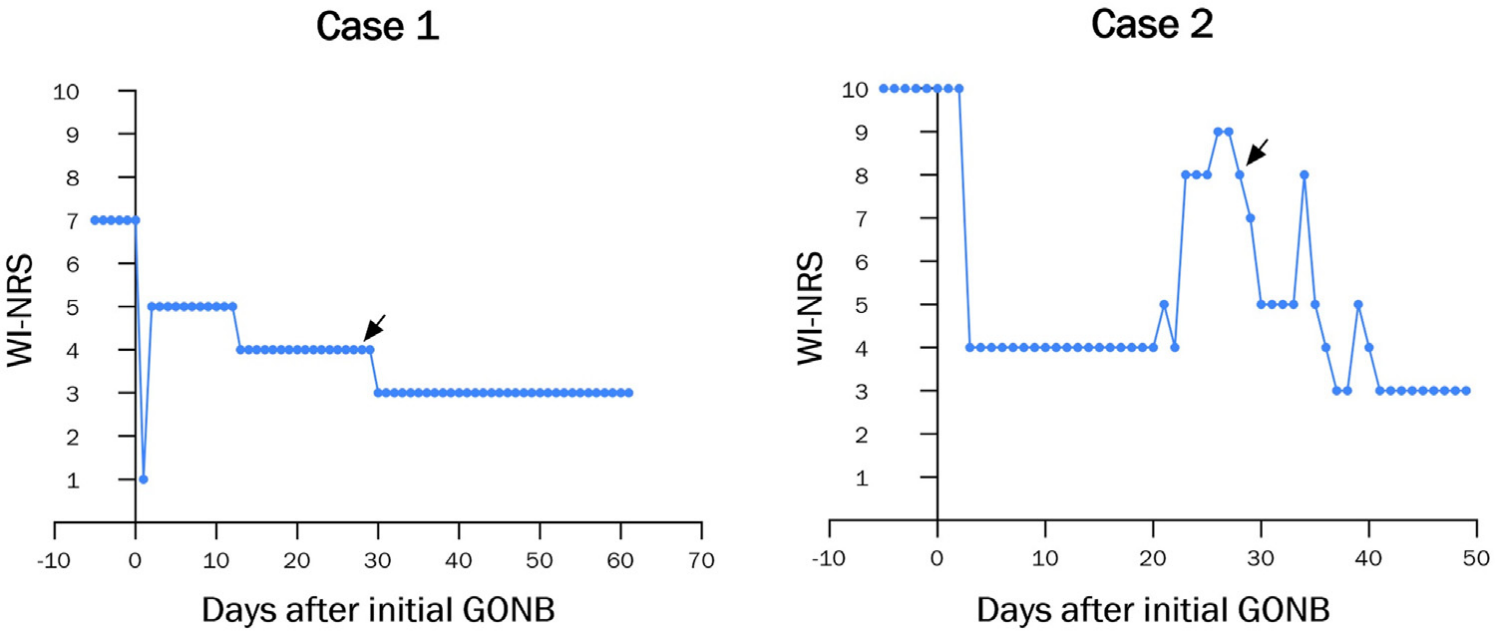
Improvement in patient-reported itch severity. Illustrated are the daily patient-reported WI-NRS scores for Case 1 (*left*) and Case 2 (*right*), beginning 5 days prior to the first GONB. Day 0 denotes the date of the first GONB, while *black arrows* indicate the date of the second GONB. *WI-NRS*, Worst Itch Numeric Rating Scale; *GONB*, greater occipital nerve block.

Due to progressively worsening quality of life (QOL), the patient opted to undergo a bedside bilateral GONB. A total of 6 mL (3 mL per side) of 0.25% bupivacaine was injected into the posterior scalp using a fanning technique at 2 injection sites, each located one-third of the distance from the occipital protuberance to the corresponding mastoid process (Fig 2). This regimen led to 4 w of sustained improvement in pruritus, with WI-NRS score down to 4/10 from his initial 7/10. The procedure was then repeated, leading to sustained itch reduction with WI-NRS score of 3/10 over the next 4 weeks (Fig 3). After repeated procedures, the patient had resolution of lesions (Fig 1, *C* and *D*). At present, he continues to receive bilateral GONB every 4 weeks, with WI-NRS score down to 2/10. He has reported no adverse effects.

### Case 2

**Fig 4:**
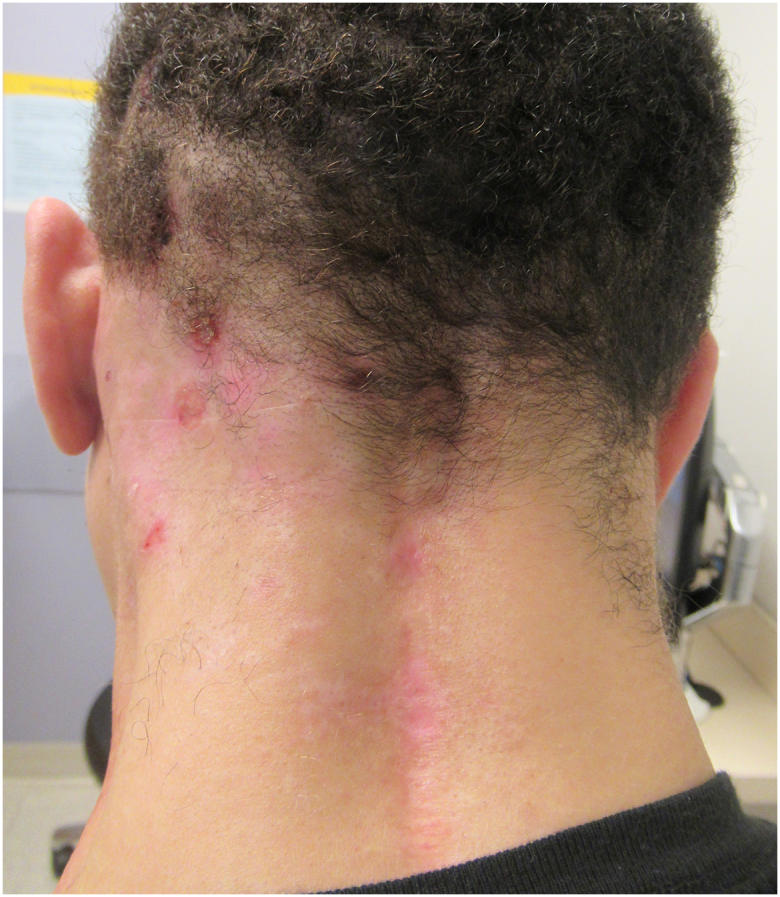
Case 2 clinical photograph. Numerous excoriations on the *left* occipital and parietal scalp.

A 39-year-old man with a history of C3-C5 fibromyxoid sarcoma status post C2-C6 posterior fusion and C2-C5 laminectomy with tumor resection presented with a 1-year history of intractable occipital scalp pruritus. WI-NRS score was 10/10. Physical examination revealed numerous excoriations on the left occipital and parietal scalp (Fig 4). Prior cervical MRI was reviewed and demonstrated diffuse dural ectasia and postsurgical changes at the C2-C6 levels. Over the next 12 months, the patient’s pruritus remained uncontrolled despite the use of numerous local and systemic agents, including intralesional steroid injections; topical corticosteroids; compounded ketamine, amitriptyline, and lidocaine cream; valacyclovir; and ketoconazole shampoo.

The patient opted to receive a bedside bilateral GONB with bupivacaine as described above (Fig 2). This regimen suppressed the patient’s pruritus to a severity of 4/10 from his initial 10/10, which was sustained for 3 weeks. The procedure was then repeated, leading to sustained itch reduction with WI-NRS as low as 3/10 over the next 3 to 4 weeks (Fig 3). At present, the patient continues to receive bedside bilateral GONB every 4 weeks, with WI-NRS score down to 0/10. He has reported no adverse effects.

## Discussion

Neuropathic pruritus results from damage to pruriceptive or itch-suppressive pathways in the peripheral or central nervous system, leading to the neuronal ability to spontaneously transmit itch. Like pain, the sensation of itch is primarily mediated by small-diameter, unmyelinated C-fibers. GONBs are commonly used to provide pain relief in various primary and secondary headache disorders such as migraine and occipital neuralgia.^6^ The greater occipital nerve, which is primarily composed of the medial branch of the C2 dorsal primary ramus, provides cutaneous innervation to most of the posterior scalp. This is a novel report describing the utilization of bedside GONB with bupivacaine in an outpatient dermatology clinic for the treatment of scalp pruritus, leading to sustained itch reduction.

Bupivacaine is a widely used local anesthetic that acts by binding to voltage-gated sodium channels and decreasing the neuronal membrane’s permeability to sodium ions, thus inhibiting depolarization and impulse conduction. It additionally inhibits NMDA receptors in the dorsal horn of the spinal cord, which are involved in central sensitization.^7^ It is thought that such effects on central pain modulation may explain the long-lasting analgesia experienced by some patients after PNB.^6^ Furthermore, bupivacaine has been shown to inhibit TRPV-1 channels.^8^ TRPV-1 is abundant in the outer root sheath of hair follicles and is implicated in skin dysesthesias and itch, including NP.^2^ Liposomal bupivacaine could also serve as a possible treatment option due to its comparable safety profile with standard bupivacaine and the capacity to provide extended relief.

Previous studies evaluating GONB in the treatment of migraine have reported a favorable safety profile. In one retrospective study of 31 patients who received GONB with 1.5 mL 0.5% bupivacaine bilaterally, 12.9% of patients reported postprocedural pain which was controlled by NSAIDs and resolved within 24 hours.^9^ No other adverse events were reported. Another trial evaluated GONB with 6 mL 0.5% bupivacaine in 51 patients. Sixteen patients reported adverse effects, including numbness, dizziness, worsening headache, injection site reaction, and gastrointestinal symptoms.^10^ A review of 14 clinical trials utilizing GONB for migraine found that side effects were reported by 39 of 600 patients and were generally mild, including nausea, vertigo, presyncope, and injection site pain or redness.^9^

This report of bedside GONB for the management of intractable scalp pruritus represents a novel treatment option for a debilitating condition with no FDA-approved therapies. Data from larger studies are needed to further evaluate the efficacy and safety of bedside PNBs in the management of scalp pruritus and other forms of NP, including scalp dysesthesia or burning scalp syndrome.

## Conflicts of interest

Dr Shawn Kwatra is an advisory board member/consultant for Abbvie, Amgen, Arcutis Biotherapeutics, Aslan Pharmaceuticals, Bristol Myers Squibb, Cara Therapeutics, Castle Biosciences, Celldex Therapeutics, Galderma, Genzada Pharmaceuticals, Incyte Corporation, Johnson & Johnson, Leo Pharma, Novartis Pharmaceuticals Corporation, Pfizer, Regeneron Pharmaceuticals, and Sanofi and has served as an investigator for Galderma, Incyte, Pfizer, and Sanofi. All other authors have no conflicts of interest to declare.
